# Establishment of a Visual Analog Scale for DBS Programming (VISUAL-STIM Trial)

**DOI:** 10.3389/fneur.2020.561323

**Published:** 2020-10-30

**Authors:** Carla Palleis, Mona Gehmeyr, Jan H. Mehrkens, Kai Bötzel, Thomas Koeglsperger

**Affiliations:** ^1^Department of Neurology, Ludwig Maximilian University, Munich, Germany; ^2^Department of Neurosurgery, Ludwig Maximilian University, Munich, Germany; ^3^Department of Translational Brain Research, German Center for Neurodegenerative Diseases (DZNE), Munich, Germany

**Keywords:** deep brain stimulation (DBS), closed loop, Parkinson's disease (PD), visual analog scale (VAS), Minkowski distance

## Abstract

**Background:** Deep brain stimulation (DBS) has become a standard treatment for advanced stages of Parkinson's disease, essential tremor, and dystonia. In addition to the correct surgical device implantation, effective programming is regarded to be the most important factor for clinical outcome. Despite established strategies for adjusting neurostimulation, DBS programming remains time- and resource-consuming. Although kinematic and neuronal biosignals have recently been examined as potential feedback for closed-loop DBS (CL-DBS), there is an ongoing need for programming strategies to adapt the stimulation parameters and electrode configurations accurately and effectively.

**Methods:** Here, we tested the usefulness of a patient-rated visual analog scale (VAS) for real-time adjustment of DBS parameters. The stimulation parameters (contact and amplitude) in Parkinson's patients with STN-DBS (*n* = 17) were optimized based on the patient's subjective VAS rating. A Minkowski distance (M_d_) was calculated to compare the individual combination of contact selection and amplitude to the stimulation parameters that resulted from classical programming based on clinical signs and symptoms.

**Results:** We found no statistically significant difference between VAS-based and classical programming in regard to the specific contact or amplitude used or in regard to the clinical disease severity (UPDRS).

**Conclusions:** Our data suggest that VAS-based and classical programming strategies both lead to similar short-term results. Although further research will be required to assess the validity of VAS-based DBS programming, our results support the investigation of the patient's subjective rating as an additional and valid feedback signal for individualized DBS adjustment.

## Introduction

Since the pioneering work of Benabid et al. (1), deep brain stimulation (DBS) has become a standard treatment for advanced stages of Parkinson's disease (PD), for medically intractable essential tremor (ET), and for complicated forms of dystonia. Apart from the careful selection of suitable patients and the correct surgical device implantation, effective postoperative programming of DBS devices is regarded as to be the most relevant factor for the individual patient outcome (2–4). Once a patient has been implanted with DBS leads, adjusting stimulation parameters is the only way to optimize the clinical effect and it becomes even more important if DBS electrodes are located outside the center of the intended target structure. DBS parameter adjustment has been shown to ameliorate patient outcomes and to prevent unnecessary lead revisions (5). In addition, a sometimes significant advancement with re-programming demonstrates that the correct adjustment of stimulation parameters is a major factor for successful treatment and patient satisfaction (6).

Despite established strategies for adjusting neurostimulation (7, 8), DBS programming requires time and personal resources. New leads with two levels of tripartite electrodes (i.e., sub-segmented electrodes) (Abbott®, Boston Scientific®) can improve the therapeutic window but increase the number of potential combinations of programming parameters (9). Therefore, there is a need for novel strategies on how to adjust stimulation parameters and lead configurations in a rapid, precise, and effective way. Currently, patient- and disease-specific biomarkers are being actively examined, which can be incorporated into adaptive closed-loop stimulation systems, responding rapidly to real-time patient needs, and avoiding the need for manual programming (10, 11). However, the most suitable biosignal remains to be determined and likely differs between different disease states and individual patients.

Here, we tested the usefulness of the patient's subjective rating as a feedback signal for DBS adjustment. We compared the specific contact and stimulation amplitude resulting from the patient's subjective rating on a visual analog scale (VAS) with a clinical standard and found no significant difference between the two programming approaches. Our results thus suggest that DBS patients are well able to adjust their IPG by themselves and will support the investigation of the patients subjective rating as a feedback signal for DBS programming.

## Methods and Materials

### Study Subjects

Study subjects were Parkinson's disease (PD) patients that had DBS of the subthalamic nucleus (STN). The study participants were recruited during routine visits at our movement disorder outpatient clinic based on their interest in participating in the study and on fulfilling the main inclusion criteria (i.e., diagnosis of an IPS and STN-DBS). All study participants fulfilled the criteria for Parkinson's Disease according to the UK Parkinson's Disease Society Brain Bank Clinical Diagnostic Criteria (12). All study participants had DBS for at least 1 year and were on a stable DBS program for at least 3 months prior to the study visit.

### Study Visit and Programming

**Figure 1:**
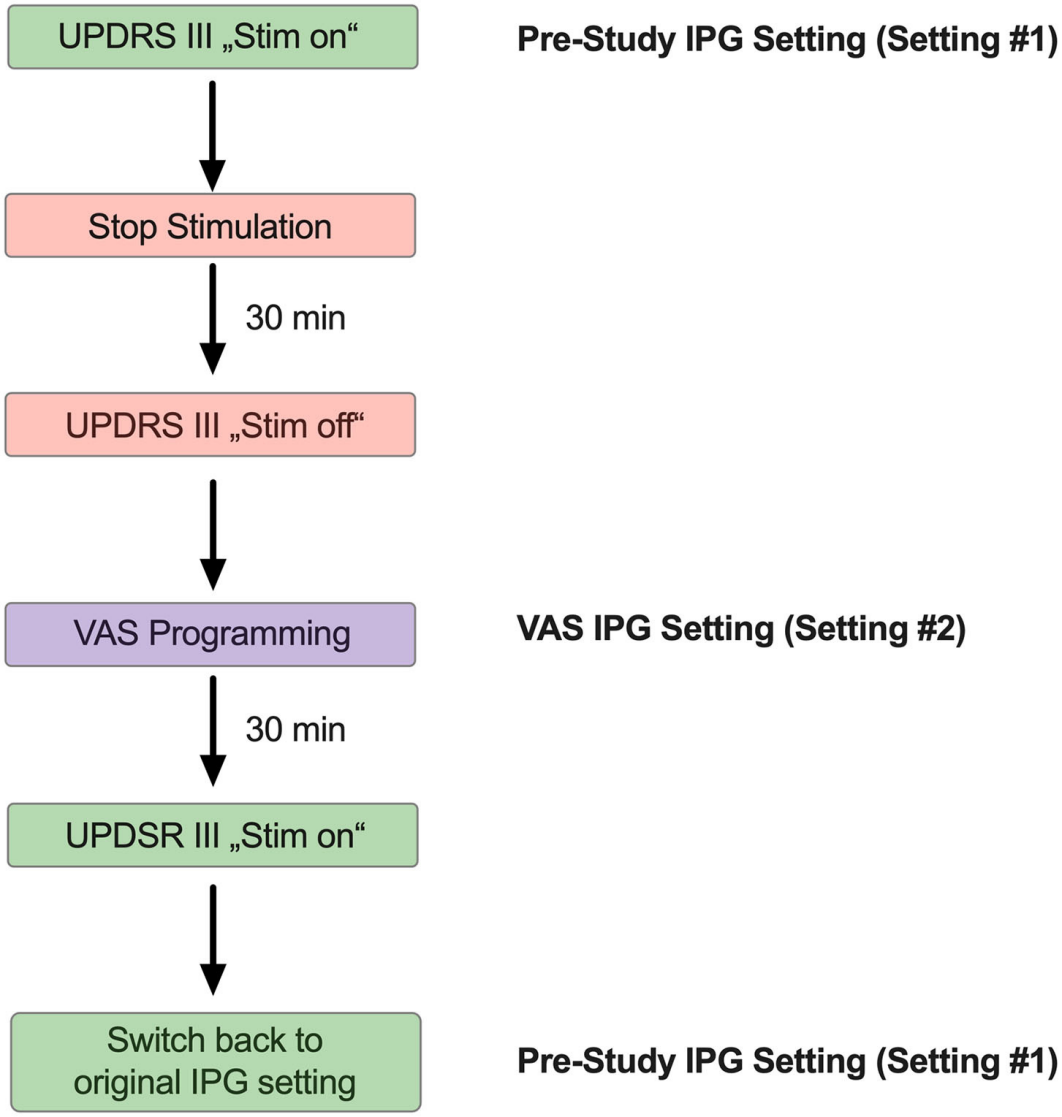
Schematic illustrating the sequential adjustment of the IPG setting during the study visit. Each study participant participated in one single study visit. At the beginning of the study visit, a UPDRS III was obtained with the stimulator switched on (“Stim on”). Next, the IPG has been switched of and the study subjects were asked to rest for 30 min, followed by another UPDRS III (“Stim off”). Thereafter, the study participants were subjected to VAS-based IPG programming. After another 30 min, a final UPDRS III has been obtained from all study subjects with the VAS-derived DBS program activated (“Stim on”). At the end of the study visit, all participants were switched back to the original setting.

In order to test the acute clinical effects of VAS-based DBS programming, we first determined the UPDRS III (“MED-ON/STIM-ON”) of all patients at the beginning of the study visit (Figure 1). All patients were examined in a pharmacological “MED-ON” state, i.e., without pausing any medication prior to the study visit. The individual patient's PD medication is specified in the Supplementary Table 1. Next, the chronic stimulation parameters were documented, the stimulation switched off on the clinically predominant side (i.e., the left electrode for the right side) and the patients asked to rest for 30 min before the UPDRS III (“MED-ON/STIM-OFF”) was measured again. Subsequently, all patients underwent a VAS-based re-programming of their stimulator on the side that has been switched off before. Therefore, the patients were presented with different amplitudes (0–3, 5 mA) on each individual contact separately. The different settings (contacts and amplitudes) were presented randomly to avoid habituation. Following each individual adjustment, the patient was asked to rate the overall quality of the DBS effect on a scale from 1 to 10. Therefore, all patients were asked “to rate the quality of the current setting on a scale from 1 to 10, where 1 is *very bad* and 10 is *very good*” without any further explanation. The values in between were not further specified or described to the patient, i.e., the rating was solely made based on the subjective perception of the patient under a given stimulation setting. The respective rating was computed and the next adjustment was made manually. All patients were blinded to the respective stimulator setting before and during adjusting the IPG. Selecting and choosing a specific VAS setting took ~15 s. After each VAS setting, a “washout” period of 60 s was maintained prior to the presentation of the next VAS setting. The overall VAS-based adjustment took ~45 min/electrode/patient

### Calculation of Minkowski Metric

A Minkowski Distance (M_d_) was calculated as a measure of similarity to compare the DBS setting before and after VAS programming. We considered the respective contact as well as the amplitude of stimulation in each patient. Analysis was conducted in R (https://www.R-project.org) and the Minkowski distance was produced using the package R stats 3.6.1 from R Studio (http://www.rstudio.com). The amplitude (mA) and the number of electrodes were adjusted for each patient in the “before” and “after” part of the formula. The maximum amplitude with clinical relevance was defined as 7 mA, as this was the maximum amplitude in our clinical cohort of patients. The maximum Minkowski value was set to 56, i.e., 4 different ring levels × 14 distinct amplitude steps (o,5-1-1,5-2-2,5-3-3,5-4-4,5-5-5,5-6-6,5-7) = 56.

## Results

### VAS-Based Programming Results in Similar Contact and Amplitude Choices

**Figure 2:**
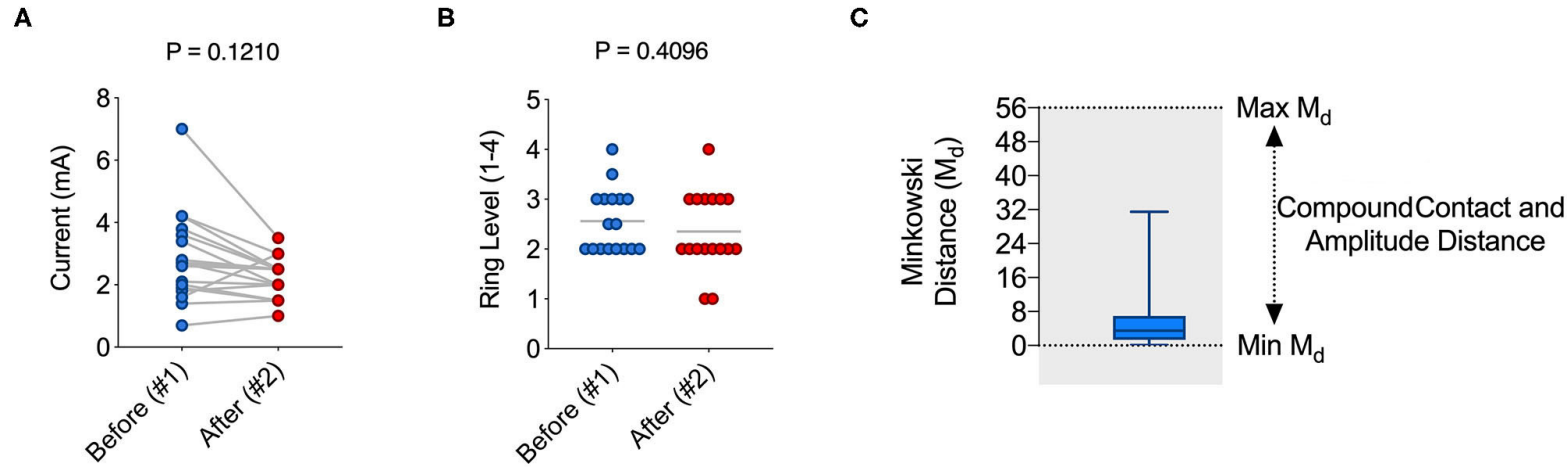
VAS-based programming leads to similar contact and amplitude choices as standard clinical programming. **(A)** Graph illustrating the current in mA chosen in setting #1 (pre-VAS) and #2 (VAS) (mean ± s.e.m: 2.853 ± 0.3552 vs. 2.235 ± 0.1553; *P* > 0.05). **(B)** Graph illustrating the ring level height (1, 2, 3, or 4) on the electrode lead in setting #1 (pre-VAS) and #2 (VAS) (mean ± s.e.m: 2.853 ± 0.3552 vs. 2.235 ± 0.1553; *P* > 0.05). **(C)** Bar graph showing the mean ± s.e.m. Minkowsi distance (M_d_) considering contact and amplitude between setting #1 and #2 (mean M_d_ ± s.e.m.: 5.49 ± 7.49). For statistical comparison, an unpaired *t*-test has been used in **(A,B)**.

We enrolled a total number of *n* = 17 PD patients (Table 1) with an average age of 61.4 ± 6,2 yrs. Disease duration was 15.76 ± 2.86 yrs with an average DBS duration of 3.29 ± 2.73 yrs. n = 3 patients had a device from Abbott® and *n* = 5 from Boston Scientific®, each of them with segmented electrodes. *n* = 9 patients had a device from Medtronic® with unsegmented electrodes. In order to compare the original stimulator settings, a result of our clinical standard programming strategies (7) (setting #1) with the VAS-based adjusted IPG setting (setting #2), we subjected each patient to a VAS-based re-programming of their stimulator, and recorded the respective VAS results for each contact and amplitude (Figure 1). This resulted in 7 distinct VAS values (o,5-1-1,5-2-2,5-3-3,5 mA) for each of the tested contacts (Supplementary Figures 1A,B) that were recorded separately. In order to avoid habituation, each patient was presented with random combinations of contacts and amplitudes. An example of sequential of IPG adjustments is illustrated in Supplementary Table 2. When we compared the selected current from our clinical standard program (setting #1) with the current from VAS-based programming (setting #2), we found no significant difference, thus indicating that on average patients chose similar stimulation amplitudes as clinicians would (Figure 2A). Likewise, we found no significant difference between the ring level heights (1–4) between the two distinct settings, thus suggesting that patients also select similar electrode levels, when following their subjective rating (Figure 2B). However, these measures represent only average differences and may not be suitable to illustrate the overall similarity between two different IPG setting. In order to generate a compound value that integrates electrode level and amplitude, we therefore calculated Minkowski distances (M_d_) for the selected contacts in setting #1 and #2, respectively (Figure 2C). In accord with our previous results, we found a very low M_d_ for both settings with 9 patients having chosen the very same electrode level (height), of which 6 patients have even chosen the same contact on segmented leads. Taken together, our results thus suggest that VAS-based programming leads to comparable results in regard to the amplitude and electrode contact as compared to our clinical standard programming.

**Table 1:** Table summarizing the demographical and device-associated characteristics of the study subjects prior to and subsequent to VAS-based DBS adjustment.

**Pat. no**.	**Age range (yrs)**	**Pre-dominant side**	**Disease onset (yr)**	**DBS duration (yrs)**	**Device**	**Frequency (Hz)**	**Pulse width (μs)**	**Type of stimulation pre-VAS (setting #1)**	**Type of stimulation post-VAS (setting #2)**	**Amplitude pre-VAS (mA)**	**Amplitude post-VAS (mA)**	**Contact pre-VAS**	**Ring level pre-VAS**	**Contact post-VAS**	**Ring level post-VAS**
1	70–75	Left	2003	1	Abbott	130	60	s	s	2,1	2,0	10/11	2/3	11	3
2	55–60	Right	2003	1	Boston	130	60	s	s	1,9	1,5	5/6/8	3/4	2	2
3	70–75	Right	2001	1	Boston	130	60	r	s	2,7	2,5	3	2	3	2
4	50–55	Left	2004	3	Abbott	130	60	r	r	7,0	3,5	10/11	2/3	11	3
5	60–65	Right	2001	4	Medtronic	130	60	r	r	3,8	2,5	1	2	1	2
6	60–65	Right	2005	5	Medtronic	130	60	r	r	1,8	2,0	1	2	1	2
7	60–65	Left	2007	1	Boston	140	60	s	s	1,4	1.5	12	2	10	2
8	55–60	Left	2002	5	Medtronic	140	60	r	r	2,7	2,0	2	3	1	2
9	55–60	Left	2014	1	Abbott	130	60	s	r	2,8	2,5	10B/10C	2	11	3
10	50–55	Left	2004	4	Medtronic	130	60	r	r	4,2	2,5	9	2	10	3
11	65–70	Left	2007	2	Boston	130	60	r	s	3,6	2,5	13/14	3	14	3
12	55–60	Right	2002	3	Medtronic	130	60	r	r	2	1,5	2	3	0	1
13	60–65	Right	2002	1	Medtronic	130	60	r	r	0,7	1,0	1	2	1	2
14	55–60	Left	2005	6	Medtronic	130	60	r	r	3,4	2,0	3	4	3	4
15	70–75	Left	1996	6	Medtronic	130	60	r	r	1,6	3,0	9	2	9	2
16	65–70	Right	2000	11	Medtronic	130	60	r	r	4,2	3,0	2	3	0	1
17	60–65	Right	2005	1	Abbott	130	60	s	s	2.6	2.5	3B	3	3B	3

*For each patient the age (yrs), the sex (m/f), the predominantly affected body side (right/left) are specified besides the time since disease onset (yrs), the time since the implantation of the DBS devise and the manufacturer. In addition, the programming details (specific contacts, stimulation amplitude, pulse width and frequency) before and after VAS-programming are specified. The type of stimulation is specified indicating either ring mode (r) or the use of subsegments on a given ring level (s). The ring level indicates the electrode height on a four-level lead (i.e., 1, 2, 3, or 4)*.

### VAS-Based Programming Results in Similar Short-Term Effects

**Figure 3:**
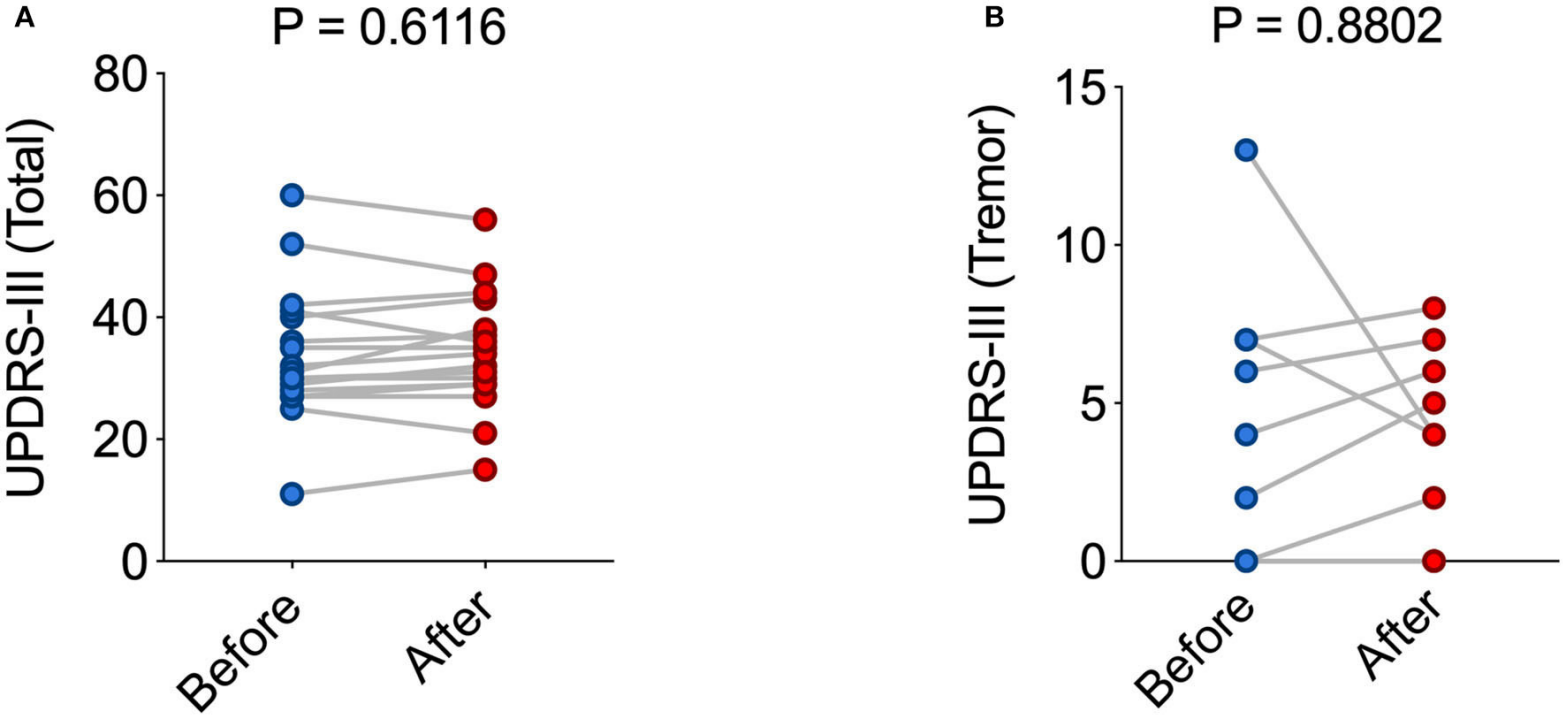
VAS-based programming leads to similar short-term clinical results as compared to standard clinical programming. **(A,B)** Graphs illustrating the total UPDRS-III value **(A)** and tremor-selective UPDRS-III value **(B)** in each individual patient with setting #1 or setting #2 (mean ± s.e.m.: 33.88 ± 2.690 vs. 34.35 ± 2.364; *P* > 0.05). For statistical comparison, an unpaired *t*-test has been used in **(A,B)**.

In order to obtain an additional clinical readout in response to VAS-based programming, we compared the UPDRS III before and after VAS-assisted programming (Figure 3). We found no statistically significant difference the total UPDRS III score (Figure 3A). Because we were addressing short-term effects and since tremor in PD in known to respond to DBS fast (7), we also compared the results of the tremor-assessing items of the UPDRS III. Similar to the total UPDRS III, we found no statistically significant difference between conventional and VAS-based programming (Figure 3B). Taken together, these results suggest VAS-based programming to result in similar short-term clinical effects as compared to our conventional approach guided by clinical signs.

## Discussion

DBS programming became more complex with the use of new leads with multi-segmented electrodes due to the exponential increase of possible combinations of programming parameters (9). Instead of the repeated application of clinical tests during programming, the patient's subjective self-raring may be just as valid as a feedback signal as clinical examination, but at the same time may be less time- and resource-consuming. In addition, such a strategy may allow for repeated re-adjustment of stimulation parameters by the patient him/herself and in the absence of regular clinical visits. This hypothesis has implications for the post-operative care of DBS patients, in particular for those who may not be able to regularly attend follow-up visits, but has never been addressed so far experimentally.

In the present work, we therefore examined the usefulness of a VAS for DBS programming in PD. We found a high degree of correlation between the stimulator setting resulting from our clinician-programmed standard approach and the setting deduced from VAS-based programming (Figure 2). Our data thus suggest that in PD, patients choose a setting for themselves that is not significantly different from the setting the experienced clinician had chosen for them. Our results agree with a recent report that supports the validity of self-assessment in DBS programming (13). Future studies are needed to compared the clinical effectiveness of the two strategies in *de novo* patients over an extended period of time.

In addition to quantifying the stimulation amplitude and ring level, we chose to calculate a Minkowski distance (M_d_) as a compound measure to compare different stimulator settings. A step size of 0.5 mA was chosen and perceived as a good compromise between accuracy and feasibility (i.e., the time necessary to repeat sequential VAS-based queries). Similar to the individual values, M_d_ values were extremely low between the two settings. Notably, one patient had pre-VAS a current of 7 mA. As we chose to test in a range of 0–3, 5 mA, the M_d_ value in this patient was higher than in the rest of the cohort, thus somewhat confounding our result. Overall, M_d_ values appear to be a reliable and useful measure to compare different IPG settings and will support the comparison of distinct programming strategies including prospective studies on closed-loop DBS.

Although the overall stimulation current was not significantly different between setting #1 and #2 (Figure 2A), there was a trend toward a lower stimulation amplitude with VAS-based programming. VAS-based programming therefore could be associated with a reduced stimulation current and thus help to prevent battery drain. However, these results need confirmation from studies that examine VAS-based settings over a longer period of time and in a larger group of patients that allows for a more robust statistical analysis.

The ability of PD patients to judge their current state of mobility correctly remains a matter of debate: some reports suggested that PD patients can provide an accurate self-report of their level of disability, even in the presence of depression and cognitive impairment (14–19). Moreover, some symptoms such as depression may even be unrecognized even by trained physicians, but were apparent on the patient's self-rating (20). Conversely, others argued against a sufficient capacity of PD patients to accurately rate their deficits (21) reminiscent of anosognosia in PD (22, 23). Poor self-awareness may correlate with axial symptoms and a longer disease duration (18). Therefore, future studies need to examine the long-term safety of VAS-based programming strategies, in particular in regard to psychiatric symptoms such as depression, anxiety or suicidality.

Our study has several important limitations: first, we compared VAS-based programming strategies against a clinical standard of patients that were stable for at least 3 months on their stimulator setting. Although these patients were programmed according to the most up-to-date DBS programming strategies (7) and by using clinical tests as a readout, the programming of these patients was not standardized, thus limiting the reliability of our comparison. Second, we here tested for the acute, short-term correlation between the two strategies (VAS vs. clinical standard). Because several DBS effects are revealed only after an extended time period (7), these clinical long-term DBS effects may not be appreciated in the present study. Notably, this applies in particular to DBS side-effects, that could limit the feasibility of self-guided DBS programming. For instance, STN-DBS has been suspected to be associated with cognitive and affective symptoms and leaving the programming to patients may encompass the rink of uncontrolled exacerbation of such side effects. Future studies should address the feasibility and—in particular—the safety of VAS-based programming strategies for the patient's long-term outcome. Finally, the patients enrolled here had already DBS at the time of enrollment. Therefore, our study participants were acquainted with the specific effects and side effects of DBS, possibly confounding the validity of our approach. Prospective trials will have to compare the different programming strategies in DBS naïve patients in order to delineate the full potential of the patient's subjective rating as a valid and safe feedback signal for DBS adjustment.

## Data Availability Statement

The raw data supporting the conclusions of this article will be made available by the authors, without undue reservation.

## Ethics Statement

The studies involving human participants were reviewed and approved by Ethikkommission bei der LMU München. The patients/participants provided their written informed consent to participate in this study.

## Author Contributions

TK and KB conceived and designed the study. CP, MG, and JM conducted the study. All authors contributed to the article and approved the submitted version.

## Conflict of Interest

The authors declare that the research was conducted in the absence of any commercial or financial relationships that could be construed as a potential conflict of interest.

## Supplementary Material

Supplementary Figure 1 Schematic illustrating the individual results from VAS-based programming in a representative patient. **(A)** First, the VAS results from each ring level (1, 2, 3, or 4) were recorded as a function of increasing stimulation amplitudes. The individual electrode contacts and amplitudes (0.5–3.5 mA) were presented randomly. In the present example, ring level 3 (i.e., contacts #13/14/15) had the best VAS ratings with a high VAS rating over the entire amplitude range (green). **(B)** Subsequently, each of the individual electrode segments were tested separately, where applicable (Boston, Abbott) and the VAS-ratings recorded for each contact. In the present example, electrode #14 had the best VAS rating with a high VAS rating over the entire amplitude range and was chosen as the “most effective” contact (green).

Supplementary Table 1 Summary of PD-associated medication taken by the study's participants. Current medication is indicated as an absolute and as L-Dopa Equivalent Dose (LED).

Supplementary Table 2 Random selection plan of the individual contacts and respective amplitudes on a segmented lead. **(A)** First, the ring level heights were randomly selected and an amplitude between 0.5 and 3.5 mA was randomly assigned to the respective ring level (blue table). **(B)** In study subjects that had segmented leads, each electrode of the tripartite ring levels was likewise selected randomly with random amplitudes (green table).
