# Supplementary figures and images for: Establishment of a Visual Analog Scale for DBS Programming (VISUAL-STIM Trial)
Source: Front Neurol. 2020 Oct 30;11:561323. doi: 10.3389/fneur.2020.561323 (PMC7661931; doi:10.3389/fneur.2020.561323)

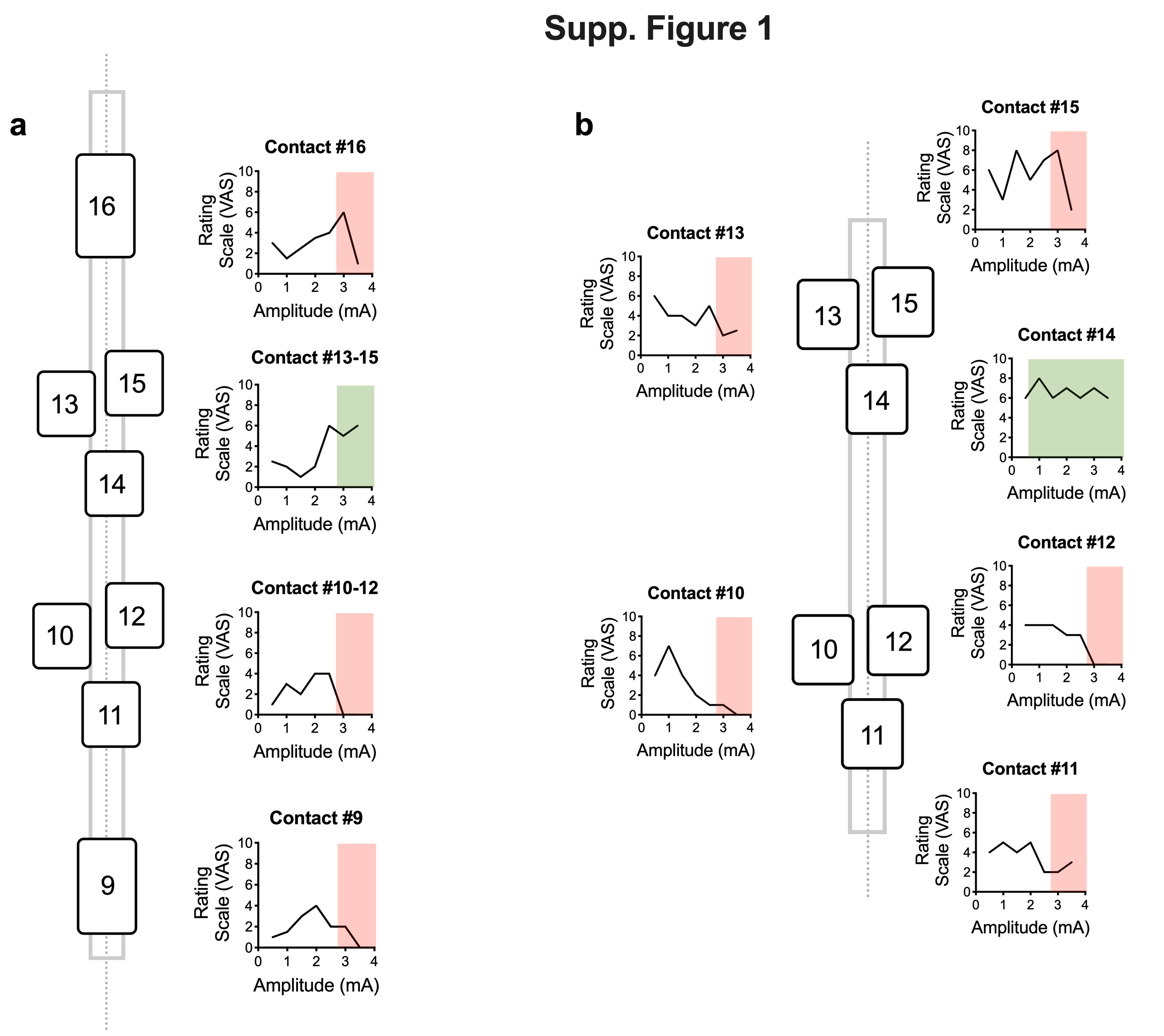

Supplement: Supplementary Figure 1 — Schematic illustrating the individual results from VAS-based programming in a representative patient. (A) First, the VAS results from each ring level (1, 2, 3, or 4) were recorded as a function of increasing stimulation amplitudes. The individual electrode contacts and amplitudes (0.5–3.5 mA) were presented randomly. In the present example, ring level 3 (i.e., contacts #13/14/15) had the best VAS ratings with a high VAS rating over the entire amplitude range (green). (B) Subsequently, each of the individual electrode segments were tested separately, where applicable (Boston, Abbott) and the VAS-ratings recorded for each contact. In the present example, electrode #14 had the best VAS rating with a high VAS rating over the entire amplitude range and was chosen as the “most effective” contact (green). [file Image_1.TIFF]
