# Supplementary material for: Establishment of a Visual Analog Scale for DBS Programming (VISUAL-STIM Trial)
Source: Front Neurol. 2020 Oct 30;11:561323. doi: 10.3389/fneur.2020.561323 (PMC7661931; doi:10.3389/fneur.2020.561323)
Supplement: Supplementary Table 1 — Summary of PD-associated medication taken by the study's participants. Current medication is indicated as an absolute and as L-Dopa Equivalent Dose (LED). [file Table_1.docx]

| **Pat. No.** | **Dopaminergic Treatment (dosage in mg/d)** | **Levodopa equivalent; LED (dosage in mg/d)** | **Motor Fluctuations After DBS Implantation** |
| --- | --- | --- | --- |
| 1 | Levodopa/Benserazid 200/50 | 200 | none reported |
| 2 | Levodopa/Benserazid 150/37,5 | 150 | freezing episodes in the evening |
| 3 | Levodopa/Benserazid 400/100 | 400 | none reported |
| 4 | Levodopa/Carbidopa/Entacapon 300/75/1200 | 400 | none reported |
| 5 | Levodopa/Benserazid 200/50 | 200 | none reported |
| 6 | Levodopa/Carbidopa 200/50, Amantadin 200, Pramipexol retard 2,1 | 610 | discrete hyperkinesia 0.5-1.5 hours after L-Dopa intake, no hyperkinesia in the DBS-OFF state |
| 7 | Levodopa/Benserazid 200/50 | 200 | freezing episodes 3-4 hours after medication |
| 8 | Pramipexol retard 1,05 retard, Levodopa/Benserazid 150/37,5 | 255 | none reported |
| 9 | none | none | none reported |
| 10 | Piribedil 50 | 50 | none reported |
| 11 | Levodopa/Benserazid 400/100 | 400 | none reported |
| 12 | Levodopa/Carbidopa 250/62,5 | 250 | none reported |
| 13 | Pramipexol retard 1,05 | 105 | none reported |
| 14 | Levodopa/Carbidopa/Entacapon 300/75/1200 | 400 | bradyhypokinesia during night time |
| 15 | Levodopa/Benserazid 600/150 | 600 | continuous bradyhypokinesia |
| 16 | Levodopa/Carbidopa 700/175 | 700 | hyperkinesia 1 hour after L-Dopa intake, no hyperkinesia in the DBS-OFF state |
| 17 | Levodopa/Benserazid 600/150 | 600 | bradyhypokinesia during night time |

**Suppl. Table 1**
