# Supplementary material for: Establishment of a Visual Analog Scale for DBS Programming (VISUAL-STIM Trial)
Source: Front Neurol. 2020 Oct 30;11:561323. doi: 10.3389/fneur.2020.561323 (PMC7661931; doi:10.3389/fneur.2020.561323)
Supplement: Supplementary Table 2 — Random selection plan of the individual contacts and respective amplitudes on a segmented lead. (A) First, the ring level heights were randomly selected and an amplitude between 0.5 and 3.5 mA was randomly assigned to the respective ring level (blue table). (B) In study subjects that had segmented leads, each electrode of the tripartite ring levels was likewise selected randomly with random amplitudes (green table). [file Table_2.docx]

| **No.** | **Contact #** | **Amplitude** |
| --- | --- | --- |
| 1 | 13_14_15 | 3 |
| 2 | 9 | 3 |
| 3 | 10_11_12 | 1,5 |
| 4 | 16 | 0,5 |
| 5 | 9 | 1 |
| 6 | 16 | 2,5 |
| 7 | 13_14_15 | 1 |
| 8 | 13_14_15 | 0,5 |
| 9 | 10_11_12 | 1 |
| 10 | 16 | 3 |
| 11 | 10_11_12 | 2,5 |
| 12 | 13_14_15 | 1,5 |
| 13 | 9 | 3,5 |
| 14 | 10_11_12 | 3,5 |
| 15 | 16 | 1 |
| 16 | 9 | 2 |
| 17 | 16 | 2 |
| 18 | 9 | 2,5 |
| 19 | 9 | 1,5 |
| 20 | 16 | 1,5 |
| 21 | 13_14_15 | 3,5 |
| 22 | 16 | 3,5 |
| 23 | 10_11_12 | 0,5 |
| 24 | 10_11_12 | 3 |
| 25 | 9 | 0,5 |
| 26 | 13_14_15 | 2,5 |
| 27 | 13_14_15 | 2 |
| 28 | 10_11_12 | 2 |

| **Nr.** | **Contact #** | **Amplitude (mA)** |
| --- | --- | --- |
| 29 | 11 | 2,5 |
| 30 | 10 | 2,5 |
| 31 | 13 | 1 |
| 32 | 13 | 2 |
| 33 | 13 | 3 |
| 34 | 10 | 2 |
| 35 | 15 | 1,5 |
| 36 | 12 | 1 |
| 37 | 10 | 3 |
| 38 | 10 | 1,5 |
| 39 | 11 | 3 |
| 40 | 11 | 2 |
| 41 | 12 | 1,5 |
| 42 | 15 | 3,5 |
| 43 | 11 | 1 |
| 44 | 13 | 2,5 |
| 45 | 15 | 2 |
| 46 | 13 | 1,5 |
| 47 | 13 | 0,5 |
| 48 | 12 | 2 |
| 49 | 12 | 3,5 |
| 50 | 10 | 3,5 |
| 51 | 12 | 2,5 |
| 52 | 14 | 2 |
| 53 | 15 | 1 |
| 54 | 14 | 2,5 |
| 55 | 14 | 1,5 |
| 56 | 14 | 3 |
| 57 | 15 | 3 |
| 58 | 10 | 0,5 |
| 59 | 14 | 0,5 |
| 60 | 10 | 1 |
| 61 | 11 | 3,5 |
| 62 | 11 | 1,5 |
| 63 | 15 | 0,5 |
| 64 | 13 | 3,5 |
| 65 | 15 | 2,5 |
| 66 | 12 | 0,5 |
| 67 | 14 | 1 |
| 68 | 12 | 3 |
| 69 | 14 | 3,5 |
| 70 | 11 | 0,5 |
| 71 | 15 | 1,5 |

**Suppl. Table 2**

**a b**
